# SCRaMbLEing to understand and exploit structural variation in genomes

**DOI:** 10.1038/s41467-018-04308-3

**Published:** 2018-05-22

**Authors:** Jan Steensels, Anton Gorkovskiy, Kevin J. Verstrepen

**Affiliations:** 1 VIB–KU Leuven Center for Microbiology, Gaston Geenslaan 1, 3001 Leuven, Belgium; 2 CMPG Laboratory of Genetics and Genomics, Department M2S, KU Leuven, Gaston Geenslaan 1, 3001 Leuven, Belgium

Structural variation is often regarded as a genetic abnormality with negative consequences. This is perhaps not surprising, since some of the best-documented instances of structural variation were detected through analysis of aberrant human cells^1,2^ and tumors^3^. However, recent analyses show that structural variation is a normal aspect of natural genetic variation. For example, a recent analysis of 2504 human genome sequences finds that structural variation is common between human individuals and population groups^4^. While it is still unclear to what extent such structural variation helped shape human evolution, experiments with microbes clearly indicate that large-scale rearrangements are an important driver of genetic adaptation to new environments (reviewed in ref. ^5^).

Interestingly, little is known about how structural variation can lead to phenotypic changes, and few studies explore the possibilities to exploit structural variation. This can be partly attributed to the use of short-read sequencing technologies, which are great at detecting SNPs and small insertions and deletions, but only offer a limited capacity to detect structural variation. Secondly, it is difficult to induce and control structural variations experimentally, impeding further investigation and exploitation.

**Fig. 1:**
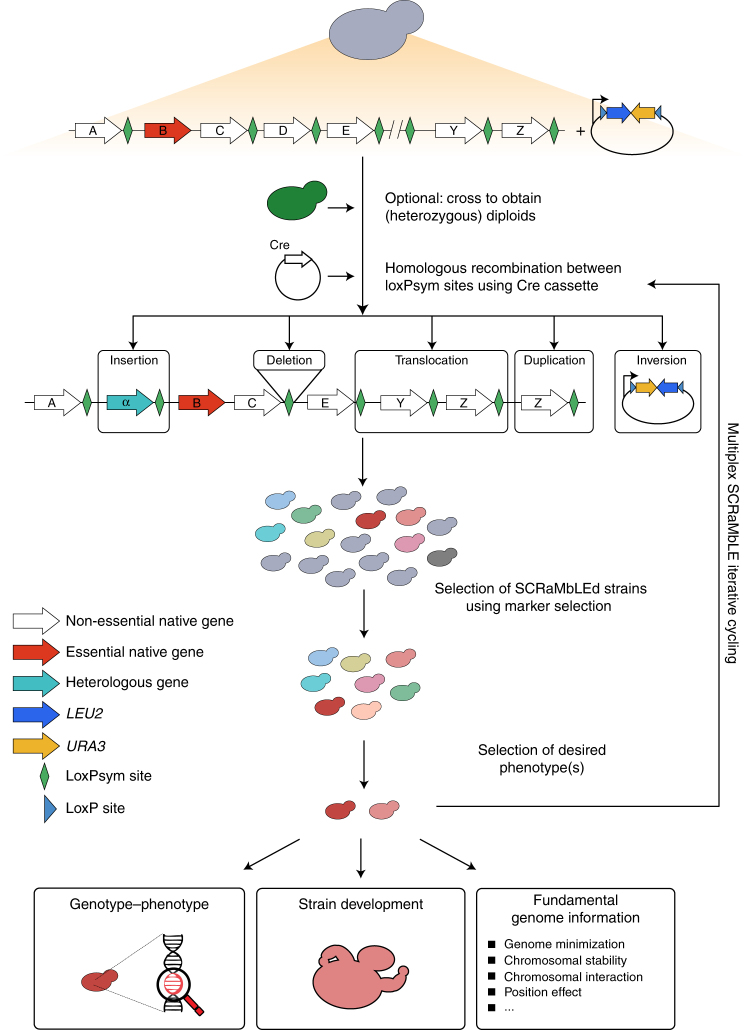
Overview of the SCRaMbLE technology and its relevance for biotechnology

Advanced synthetic biology allows inducing structural variation in genomes. The technology, appropriately called “Synthetic Chromosome Recombination and Modification by LoxP-mediated Evolution” (SCRaMbLE), makes it possible to rapidly induce recombination events that lead to extensive rearrangements between specific sites. SCRaMbLEing genomes does not only allow investigating if and how gross rearrangements lead to phenotypic changes, but also opens new avenues to create superior (micro)organisms (Fig. 1).

SCRaMbLE was first introduced as part of the Sc2.0-project, where an international team of researchers aims at synthesizing the entire *Saccharomyces cerevisiae* genome. While the Sc2.0 project largely kept the natural genomic DNA sequence, a few notable changes were introduced. One of these changes was the insertion of site-specific recombination sites, called loxPsym sites, 3 bp downstream of the stop codon of every non-essential gene, as well as near chromosomal landmarks like the centromere and telomeres. Like the natural loxP sites derived from bacteriophage P1, loxPsym sites promote direct recombination by the Cre recombinase^6,7^. However, in contrast to the natural sites, loxPsym sites facilitate recombination in either direction, which vastly expands the spectrum of potential rearrangements^8^. To ensure fast (de)activation and low leaky expression of the recombinase enzyme, several modified Cre expression vectors were developed^9^. Together, the combination of strategically placed recombination sites and tightly controlled Cre activity allows inducing a temporary burst in structural variation.

The first demonstration of SCRaMbLE was published in 2011^9^. Activating the recombinase in strains carrying a partly synthetic chromosome with 43 loxPsym sites resulted in mutant lines that showed a large variation in growth rate. Deep sequencing of 64 SCRaMbLEd mutants of a strain containing a circular synIXR chromosome revealed a unique pattern of on average 6.2 complex recombination events in each mutant^10^. Importantly, recombination events were limited to loxPsym sites, with the rest of the genome left untouched.

SCRaMbLE allows investigating how structural variation can lead to phenotypic changes. One way to do this will be to grow pools of variants in different stressful environments. Variants showing improved fitness can easily be enriched by prolonged exposure to the stress, and long-read whole-genome sequencing allows determining the structure of the recombined genomes. This enables researchers to study the phenotypic effect of genetic perturbations that are difficult to obtain using “traditional” genetic engineering strategies (inversions, duplications, translocations, etc.), and will thus further expand our knowledge of complex phenotypes.

However, there are still some pitfalls. Firstly, rearrangements are difficult to re-engineer one by one, making it difficult to distinguish the rearrangements that are associated with the change in fitness from other, neutral or slightly detrimental rearrangements. A second, and perhaps more important pitfall is that the rearrangements obtained through SCRaMbLE only occur between loxPsym sites and may therefore not be a perfect representation of naturally occurring events. Moreover, it is also still unclear if and how the rearrangements are influenced by the relative positioning of the loxPsym sites. Thirdly, deleting or amplifying certain genes can be lethal, causing recombination biases that can stretch over several kilobases. Lastly, SCRaMbLE is limited to strains harbouring the loxPsym sites, which is currently only the S288c lab strain.

It is easy to imagine how SCRaMbLE could be exploited to fine-tune, prototype or even discover new complex pathways for many biotechnological applications. For example, while the production of artemisinic acid in yeast is arguably the greatest achievement in fungal synthetic biology thus far, it was estimated to have required roughly 100 postdoc years^11,12^. Theoretically, engineering such complex pathways could be sped up drastically with clever design of loxPsym-containing neochromosomes, or recombination-mediated integration of LoxPsym-flanked DNA containing required genes. Since gene expression depends on the chromosomal location^13^, this approach can be applied for combinatorial optimization of pathway gene expression levels. Moreover, since the loxP-mediated recombination system is not specific for *S. cerevisiae*, the general concept of SCRaMbLE can be extrapolated to other industrially relevant yeasts and bacteria^14,15^. As the cost of synthetic DNA keeps falling, and the availability of CRISPR-Cas9-based strategies for rapid and efficient integration of heterologous DNA, it seems likely that other industrially relevant organisms will be equipped with recombination sites to perform SCRaMbLE.

Taken together, it is clear that SCRaMbLE is a promising new way to obtain superior microbes for industrial applications. Large pools of genetic variants differing in gene content and genome structure can be obtained in just a matter of days and, thus, parallel optimization of complex multigenic traits can be performed. The technology complements the existing toolbox, from classic breeding, mutagenesis and genome shuffling over directed evolution to genetic engineering^16^. Importantly, the technology is orthogonal to these existing methods, as the type of changes that SCRaMbLE induces is fundamentally different. Hence, it seems interesting to combine SCRaMbLE with other techniques.

That said, there are also limitations. Firstly, the dependence on loxP or loxPsym sites implies that recombination is not completely random and that the recombination sites need to be carefully chosen. Moreover, as SCRaMbLE results in extremely large pools of genetically diverse mutants, its applicability for industrial strain development depends on whether the phenotype of interest is easily selectable for. Therefore, finding clever strategies to detect improved variants, even for “difficult” phenotypes like production of specific metabolites, is vital. Lastly, it is important to realize that structural variation is sometimes seen as a “quick and dirty” way to rapidly optimize a phenotype. Other mutation events, including smaller rearrangements and changes in the DNA sequence may be needed to further optimize the phenotype under selection without scarifying other desirable traits. Still, SCRaMbLE is clearly more than a gimmick, and it will be exciting to see how the technology will help us to develop novel drugs, biomaterials or chemicals, and reach higher productivity, quality and sustainability in various industries.
